# The Effects of Electrical Stimulation on a 3D Osteoblast Cell Model

**DOI:** 10.3390/cells14060396

**Published:** 2025-03-08

**Authors:** Crystal O. Mahadeo, Alireza Shahin-Shamsabadi, Maedeh Khodamoradi, Margaret Fahnestock, Ponnambalam Ravi Selvaganapathy

**Affiliations:** 1 Neuroscience Graduate Program, McMaster University, Hamilton, ON L8S 4K1, Canada; mahadeco@mcmaster.ca; 2 School of Biomedical Engineering, McMaster University, Hamilton, ON L8S 4L8, Canada; a.shahin88@gmail.com (A.S.-S.); khodamom@mcmaster.ca (M.K.); 3 Department of Mechanical Engineering, McMaster University, Hamilton, ON L8S 4L8, Canada; 4 Department of Psychiatry and Behavioural Neurosciences, McMaster University, Hamilton, ON L8S 4K1, Canada

**Keywords:** 3D in vitro model, 3D bone construct, bone tissue, electrical stimulation, hydroxyapatite, SaOS-2 cells, human osteosarcoma cells, osteoblasts, calcium, osteocalcin, alkaline phosphatase

## Abstract

Electrical stimulation has been used with tissue engineering-based models to develop three-dimensional (3D), dynamic, research models that are more physiologically relevant than static two-dimensional (2D) cultures. For bone tissue, the effect of electrical stimulation has focused on promoting healing and regeneration of tissue to prevent bone loss. However, electrical stimulation can also potentially affect mature bone parenchymal cells such as osteoblasts to guide bone formation and the secretion of paracrine or endocrine factors. Due to a lack of physiologically relevant models, these phenomena have not been studied in detail. In vitro electrical stimulation models can be useful for gaining an understanding of bone physiology and its effects on paracrine tissues under different physiological and pathological conditions. Here, we use a 3D, dynamic, in vitro model of bone to study the effects of electrical stimulation conditions on protein and gene expression of SaOS-2 human osteosarcoma osteoblast-like cells. We show that different stimulation regimens, including different frequencies, exposure times, and stimulation patterns, can have different effects on the expression and secretion of the osteoblastic markers alkaline phosphatase and osteocalcin. These results reveal that electrical stimulation can potentially be used to guide osteoblast gene and protein expression.

## 1. Introduction

Bone is a complex and dynamic tissue with substantial capacity to regenerate itself after extensive damage [1]. It not only plays a major role in physical support, locomotion, and structure, but also is an endocrine tissue that can affect the rest of the body by regulating the body’s metabolism, renal, neural, and cardiovascular functions, and even embryonic development via secretion of different endocrine factors [2,3]. These functions are particularly important, as it has become evident that bone’s natural structure and function are affected by physical activity and exercise [4]. Determining how physical activity affects bone tissue and cells is a pivotal step in understanding overall human health, making physiologically relevant in vitro models of bone necessary for studying this tissue.

Most studies in bone biology use two-dimensional (2D) in vitro models, but it has been shown in other tissue models that models with three-dimensional (3D) microstructural properties and dynamic stimuli are better representatives of in vivo conditions [5,6]. Newer biofabrication techniques, capable of recreating important in vivo features such as 3D cell–cell and cell–extracellular matrix (ECM) interactions and dynamic cues, mimic structural and functional characteristics of different tissues [7]. These models can also be utilized to study bone tissue.

Although tissue engineering methods have been used to develop physiologically relevant in vitro models, initial work in the field was mostly aimed at regenerating lost or damaged bone due to injury or other pathological conditions such as cancer. Scaffolds that promote cell–cell and cell–scaffold interactions, sometimes with electrical stimulation to further boost cellular remodeling, constitute one of the most promising approaches investigated [8]. Biophysical stimulation is a commonly used non-invasive orthopedic therapy that uses electrical stimulation to increase repair and anabolic processes in tissue. Electrical stimulation alone has also been shown to promote healing and influence stem cell differentiation into bone-forming cells (osteoblasts). Many of the other functions of osteoblasts are affected by electrical stimulation as well, including their proliferation, migration, and bone formation (i.e., ECM deposition and mineralization) [9]. Additionally, bone exhibits piezoelectricity that converts mechanical stress to electrical signals that regulate bone cell gene expression [10]. Since bone is an electrically active and piezoelectric material, mechanical stresses on the bone due to exercise, or even less intensive movement of any kind such as walking, can generate local electrical charges and stimulation through mechanotransduction. This local electrical stimulation has the potential to influence gene expression and signaling factor production. Interpretable models that are easily controlled can recreate the dynamic effect of physical activity on the bone to help us understand the paracrine and endocrine effect of bone expressed hormones. In order to develop such a model, understanding the effects of electrical stimulation on the expression of crucial hormones and molecules in 3D bone tissue are needed. Additionally, it is unclear if electrical stimulation applied to a 3D bone construct can induce changes that are consistent with physical activity.

Electrical stimulation in vitro creates a dynamic microenvironment for cells in 2D and 3D culture systems. For example, an electric field can promote migration, proliferation or differentiation of rat and human mesenchymal stem cells, depending on conditions of the stimulation and often measured by monitoring osteogenic proteins such as alkaline phosphatase [11,12,13,14,15,16]. Osteoblast cells are derived from mesenchymal stem cells, and during their differentiation process, once progenitor cells are committed to the osteoblast lineage, they exit the cell cycle and secrete collagen and alkaline phosphatase into the extracellular environment. Alkaline phosphatase and osteocalcin, two of the major proteins synthesized by osteoblasts, are used as markers of osteogenic maturation [17,18] and to evaluate the level of differentiation to an osteoblastic phenotype [19,20]. Bone-derived alkaline phosphatase is an early osteogenic marker whose levels increase at the beginning of osteogenic cell differentiation [21]. Alkaline phosphatase is essential for bone mineralization regulation, as it hydrolyzes pyrophosphate which inhibits mineralization, osteoid formation (the matrix of bone), and calcification [22]. Interestingly, while many cells express alkaline phosphatase, osteoblasts are the only cell type that secretes osteocalcin, a protein that regulates mineral deposition [23].

Osteocalcin is a late osteogenic marker of osteoblasts that typically exhibits a high affinity towards bone ECM components such as hydroxyapatite and is a known regulator of bone mineralization [18]. Osteoblasts are bone-forming cells, the only cells that make osteocalcin. High osteocalcin levels are an indicator of bone formation. When the environmental pH drops, osteocalcin becomes decarboxylated and is released into the circulation [3]. While both carboxylated and uncarboxylated osteocalcin are found in the circulation, uncarboxylated osteocalcin serves major endocrine functions via its involvement in exercise capacity, glucose homeostasis, and brain development and cognition [24]. For example, osteocalcin promotes the secretion of insulin by the pancreas [25] which is related to high energy demands needed for bone’s constant remodeling and healing of fractures and for muscle’s energy consumption during physical activity [24]. Osteocalcin also affects adipose tissue metabolic pathways by increasing adiponectin expression and the total number of adipocytes and fat mass [26]. Not only do osteocalcin levels increase during exercise and play a major role in adaptation to physical activity [27], but, coinciding with diminished capacity with aging, its levels decline in older adults [28]. Understanding how alkaline phosphatase and osteocalcin levels change in the body is necessary to our knowledge of bone as an endocrine organ and to our overall health. Despite its importance and relevance, the effect of electrical stimulation on osteocalcin and alkaline phosphatase protein and RNA levels in 3D osteoblast cell constructs has not been investigated.

The aim of this study is to examine how electrical stimulation affects expression levels of markers of osteogenic differentiation in SaOS-2 osteoblast-like 3D cell constructs using a biofabricated in vitro and dynamic model of bone tissue. Cells in a 3D collagenous environment are treated with electrical stimulation to mimic an in vivo-like, dynamic environment. Different stimulation conditions are applied to the cells, and their response is studied by measuring the expression and secretion of alkaline phosphatase and osteocalcin. The importance of the inorganic phase of bone and its effect on gene expression is also studied by including hydroxyapatite, a major component of bone, along with the collagen matrix. We propose that the protein and gene expression of bone biomarkers osteocalcin and alkaline phosphatase can be influenced by the application of different types of electrical stimulation in this 3D model, and that this system can provide insights into the role that bone tissue plays in maintaining the health and function of other tissues.

## 2. Materials and Methods

### 2.1. Cell Culture

All reagents were obtained from Thermo Fisher Scientific (Burlington, ON, Canada) unless otherwise indicated. Osteoblast-like cells from the SaOS-2 human osteosarcoma cell line were chosen for this study because they exhibit a mature osteoblastic phenotype when compared with other commonly used cell lines such as MG-63 and U-2 OS [29]. Cells were cultured at 37 °C with CO_2_ at 5% and 95% humidity in cell culture medium consisting of McCoy’s 5A medium supplemented with 15% fetal bovine serum (FBS) and 1% penicillin–streptomycin 100X until 70% confluent. Once 70% confluent, all medium was removed, and cells were then washed with phosphate-buffered saline (PBS) and incubated with trypsin for 3 min at 37 °C. Once cells were lifted from the dish, the trypsin was neutralized using 5 mL of growth medium per plate, and cells were collected in a tube and centrifuged at 125× *g* for 5 min. Cells were then used for biofabrication of 3D cellular constructs.

### 2.2. Biofabrication of 3D Cellular Constructs

**Figure 1:**
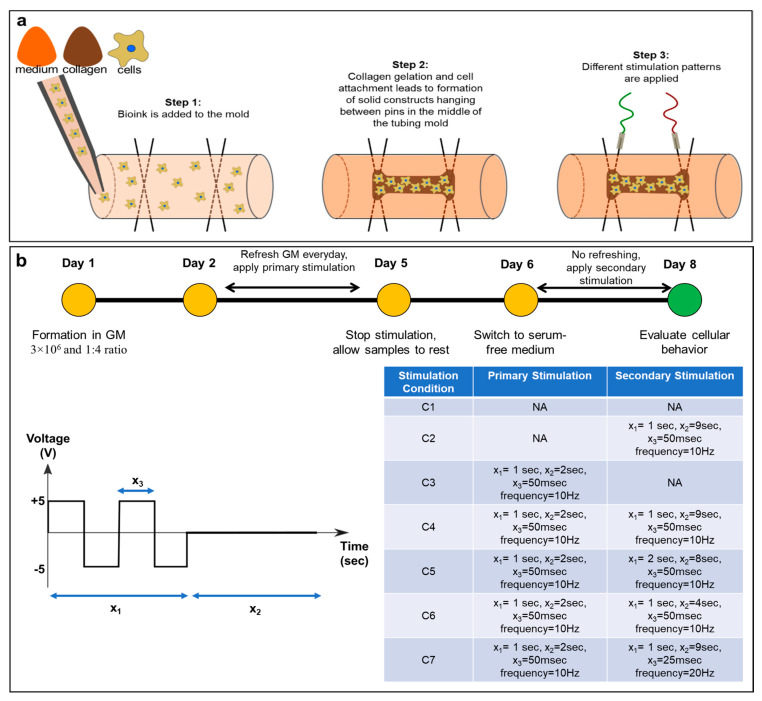
(**a**) Schematic of the biofabrication process and (**b**) culture conditions of the SaOS-2 3D constructs formed using bioink with a cell density of 3 × 10^6^ cells/mL and collagen-to-medium ratio of 1:4 maintained in static condition 1 (C1) or 6 different dynamic culture conditions (C2–C7) explaining primary and secondary stimuli. The frequency, determined by the wave and pulse width, for all primary or secondary stimulations with an x_3_ = 50 ms was 10 Hz, and those with an x_3_ = 25 ms was 20 Hz. GM: growth medium. x_1_ total duration of stimulation, x_2_ total rest time, x_3_ pulse width.

3D cellular constructs were formed using a previously developed technique called tissue-in-a-tube [30]. A pair of stainless steel pins (Bend-and-Stay 304 Stainless Steel Wire, McMaster-Carr, cat. no. 6517K11, Elmhurst, IL, USA), inserted perpendicularly into oxygen permeable silicone tubing (inner diameter of 3 mm, Sigma-Aldrich, Oakville, ON, Canada) 2 cm apart at each end as anchors, was used as the mold (Figure 1a). This mold was filled with 250 μL bioink which contained 3 × 10^6^ cells/mL and a 1:4 ratio mixture of 5 mg/mL bovine collagen type I and cell culture medium. Constructs in all groups were formed using the same number of cells which result in the same size and thickness of constructs for all experiments [31]. To prepare the bioink, collagen solution and cell culture medium were mixed, and the solution was neutralized with sterile-filtered 0.1 M NaOH before cells were added. The bioink was added to the mold and incubated at 37 °C for 2 h to form stable 3D constructs (Figure 1a).

### 2.3. Dynamic Culture Conditions

Medium was refreshed one day after construct formation, and constructs were maintained in culture with daily changes in medium under either static conditions (no electrical stimulation) or dynamic conditions with primary stimulation (Figure 1b). Primary electrical stimulation was applied for 3 days and was a square wave with a high period of 50 ms and peak-to-peak voltage of −5 to +5 V, applied with stimulation–rest ratio of 1:2 s. After one day of no stimulation for all of the groups to allow them to rest, medium was switched to nucleoside-free, serum-free α-MEM, and cells were exposed to either static culture or one of four different secondary dynamic stimulation conditions. Secondary electrical stimulation was applied for 2 days and was a square wave with a peak-to-peak voltage of −5 to +5 V and either high period and stimulation–rest of 50 ms and 1:9 s, 50 ms and 2:8 s, 50 ms and 1:4 s, or 25 ms and 1:9 s (Figure 1b). Secondary stimulation was continued for two days without refreshing the medium, and at the end, conditioned medium as well as the constructs were collected and used for further analysis. These stimulation times were selected because longer stimulation periods resulted in necrosis. A short rest period was incorporated to ensure cell constructs would not overheat and burn. Similarly, bone cells adapt to mechanical loading during exercise, but they can also become insensitive to the load very quickly. Recovery time between loading sessions allows cells to regain their mechanosensitivity [32]. Therefore, considering different stimulation and rest times in in vitro models is pivotal for developing models that can accurately model different types of activity.

### 2.4. Biochemical Analysis of Bone Factor Secretion

Levels of osteocalcin and alkaline phosphatase were measured in the conditioned medium in order to study the effects of different stimulation conditions. At the end of the secondary stimulation process, conditioned medium from each construct was collected and centrifuged to remove cellular debris. Medium was frozen at −20 °C for further use. A human osteocalcin ELISA kit (Thermo Fisher Scientific) and a colorimetric alkaline phosphatase (ALP) assay kit (Abcam, Waltham, MA, USA) were used according to the manufacturer’s instructions. Conditioned medium was used for ELISA without any dilution, while for the ALP assay a 1:200 dilution was required. Each sample was run in duplicate, and the average was used for further analysis. This experiment was repeated three times.

### 2.5. ECM Composition

Constructs were formed as described above, but hydroxyapatite particles < 200 nm particle size, ≥97% (Sigma-Aldrich) were added to the bioink to a final concentration of 1 mg/mL. Constructs were maintained under dynamic culture with both primary and secondary electrical stimulation (C4 culture condition), and the levels of osteocalcin were measured in duplicate on three samples and compared to three samples without hydroxyapatite cultured using the same dynamic condition.

### 2.6. Gene Expression

RNA was extracted from approximately 1 × 10^6^ SaOS-2 cells/sample using 1 mL TRIzol^®^ and the Qiagen RNeasy Plus Micro Kit (Qiagen, Toronto, ON, Canada) or the PureLink RNA Micro Kit. Briefly, samples were sonicated in Trizol^®^ using a Sonic Dismembrator Model 100 (Fisher Scientific, Ottawa, ON, Canada), and 200 µL of chloroform was added. Samples were centrifuged for 15 min at 11,290× *g* at 4 °C, the aqueous (RNA containing) phase was removed, and 70% ethanol was added at a 1:1 V/V ratio. Samples were applied to the silica membrane and treated with either RNase-Free DNase (Qiagen) or ezDNase enzyme (Thermo Fisher Scientific) for 15 min. RNA was eluted in RNase-free water following the manufacturer’s protocols. RNA concentration and purity were determined using a Multiskan GO spectrophotometer and SkanIT software version 3.2.1.4 (Thermo Fisher Scientific).

Approximately 500 ng of RNA in a 20 µL reaction was reverse transcribed in a GeneAmp PCR system 2400 thermal cycler (Applied Biosystems, Mississauga, ON, Canada) using the SSIVVILO kit (Thermo Fisher Scientific) following the manufacturer’s protocol. “No-RT” negative controls contained water instead of enzyme. Quantitative real-time polymerase chain reaction (qPCR) primers were synthesized by Integrated DNA Technologies Inc. (IDT) (Coralville, IA, USA). Primer sequences and product sizes are shown in Table 1. For all qPCR reactions, primers were mixed with water and PowerTrack SYBR Green Master Mix. qPCR was performed in a QuantStudio 3 (Applied Biosystems) running QuantStudio design and Analysis software version 1.5.1. Three technical replicates were run for each sample, and the plates included no-RT negative controls and a standard curve for absolute quantitation. To ensure that there was no master mix or plate contamination, no-template controls that contained qPCR master mix and water in place of samples were also included. Thermal profiles for *human alkaline phosphatase* and *human osteocalcin* were as follows: 2 min at 50 °C, 10 min at 95 °C, and 45 cycles of 10 s at 95 °C, 3 s at 65 °C and 12 s at 72 °C. This was followed by a dissociation curve of 15 s at 95 °C, 60 s at 60 °C and 15 s at 95 °C. Targets were normalized to *GAPDH*. The thermal profile for *human GAPDH* was 2 min at 50 °C, 2 min at 95 °C, 40 cycles of 15 s at 95 °C and 60 s at 60 °C, followed by a dissociation curve 15 s at 95 °C, 60 s at 60 °C and 15 s at 95 °C. The mean of triplicates was used for further analysis.

### 2.7. Phalloidin Staining and Microscopy

**Figure 2:**
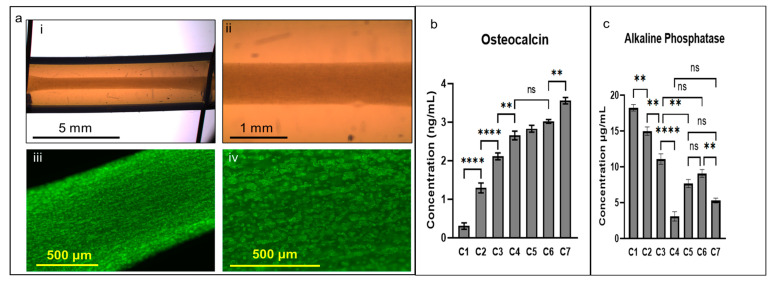
Electrical stimulation regimen affects protein expression. (**a**) Three-dimensional cellular constructs and cell distribution (low- and high-magnification): (i,ii) bright field microscopy of constructs) and (iii,iv) fluorescence images of phalloidin stained constructs. Effect of different stimulation conditions on (**b**) osteocalcin and (**c**) alkaline phosphatase secretion. ** *p* < 0.01, and **** *p* < 0.0001, ns not significant. Error bars represent SEM. One-way ANOVA, post hoc Tukey multiple comparisons. This experiment was performed 3 times for a total n of 12/group, with each sample assayed in duplicate.

At the end of each experiment, constructs were extracted from the tubing and fixed in a 2% wt/vol formaldehyde solution for 30 min. After washing 4 times with phosphate-buffered saline (PBS), constructs were stained with Phalloidin solution (Alexa Fluor™ 488, Invitrogen, Burlington, ON, Canada) for 1 h using 1 mL of PBS containing 1 μL of stock solution of Phalloidin (300 units dissolved in 1.5 mL methanol). Samples were washed in PBS and imaged using an inverted fluorescent microscope (Olympus IX51, Olympus, Richmond Hill, ON, Canada) with a green dichroic fluorescence filter (FITC) with 475–495 nm excitation and 512–536 nm emission. Images of the stained constructs were captured using a digital monochromatic greyscale Qimaging Retiga 2000R camera (QImaging, Surrey, BC, Canada). Imaging was also performed while constructs were still in the tubing using a dissecting microscope (Olympus, Richmond Hill, ON, Canada) with 0.8× and 2× magnifications (Figure 2a(i–iv)).

### 2.8. Alizarin Red Staining and Microscopy

At the end of the experiment, constructs were fixed in the tubing using a 2% wt/vol formaldehyde solution for 30 min. Constructs were then rinsed 4 times with PBS. Constructs were removed from the tubing and placed in a 6-well plate. Alizarin red (2% wt/vol) (Sigma-Aldrich, Oakville, ON, Canada) was dissolved in deionized water, mixed and filtered and 1 mL was added to each well. Constructs were incubated in stain for 10 min and then washed with PBS 5 times. Constructs were imaged using an EVOS Invitrogen XL core imaging system. A custom python code was used to analyze the images for redness. Images were first converted to grayscale and an intensity threshold was applied. HSV (hue, saturation, and value) was used to analyze the colored regions of the samples in the images. The redness was calculated by using these three factors. Specifically, the hue angle, saturation level, proximity to redness (low and high hue values), and brightness were used to calculate a redness score on a 0–100 scale (higher values equaling higher red intensity).

### 2.9. Scanning Electron Microscopy (SEM) and Energy-Dispersive Spectroscopy (EDS)

SaOS-2 3D constructs were dehydrated using a graded ethanol series (50%, 70%, 70%, 95%, 95%, 100%, 100%) for 10 min each. The samples were then kept in 100% EtOH, and placed into a Leica EM CPD300 critical point dryer (Leica Mikrosysteme GmbH, Wien, Austria) for dehydration. The chamber was sealed and then flushed 12 times with liquid CO_2_. The CO_2_ filled chamber was heated to 35 °C and pressure increased to above 1100 psi for the CO_2_ liquid phase to gaseous phase conversion. The gas was vented slowly from the chamber. Dried constructs were mounted onto SEM stubs with double-sided carbon tape and then placed in the chamber of an Edwards sputter coater to be coated with 15 nm of gold. The samples were viewed in a Vega II LSU scanning electron microscope from Tescan USA (Warrendale, PA, USA) operating at 20 kV. The SEM was equipped with an Oxford X-Max 80 energy-dispersive spectroscopy detector and Inca software version 4.14 from Oxford Instruments (Abingdon, UK) from which spectra and weight percentages were obtained.

### 2.10. Statistical Analysis

Statistical analyses were performed using GraphPad Prism software 8.0.2. One-way analysis of variance (ANOVA) with the post hoc Tukey multiple comparisons test or Dunn’s test was used for group comparisons, with *p* < 0.05 set as statistical significance. Student’s *t*-test was used for comparisons between two groups. For non-parametric data, a Kruskal–Wallis test with a post hoc Dunn test was used for group comparisons with *p* < 0.05 set as statistical significance. Groups were treated independently for post hoc analysis, and meaningful significant differences and data were reported as mean values ± SEM. *p* < 0.05, *p* < 0.01, *p* < 0.001, and *p* < 0.0001 are shown as *, **, ***, and ****, respectively.

## 3. Results

### 3.1. Electrical Stimulation of 3D Osteoblast Cell Culture Alters Protein Expression

A 3D model of human bone tissue was fabricated using the human SaOS-2 osteosarcoma cell line and a previously developed biofabrication technique [30]. Electrical stimulation was applied to create a dynamic environment for the cells, and its effect on cellular protein expression was studied. The bone constructs were exposed to different stimulation conditions (Figure 1b) with different frequency oscillations based on a range previously used in other studies. Studies examining the effects of electrical stimulation on bone healing, regeneration and remodeling used electrical stimulation frequencies between 0 and 100 Hz [33,34]. For our study, higher frequencies (50–100 Hz and higher) were avoided because, when tested, they overheated and burned the constructs with consequent degradation of the cells as determined by RNA quality and yield. The effect of electrical stimulation on protein expression was assessed by measuring the bone markers osteocalcin and alkaline phosphatase. To understand the effect of stimulation timing and its long-term effects on the cells, cellular constructs were exposed to two separate bouts of stimulation over the course of 8 days. The initial or “primary” stimulation was conducted for 3 days followed by a day of rest, and a follow-up (secondary) course of stimulation was conducted for 2 days. This was to ensure that no burning of the constructs would occur and to reflect changes in physical activity with pauses in between. Lastly, secondary stimulation conditions with different total rest times (x_2_) (9, 8, and 4 s) were tested to examine the effect of shorter rest times on the amount of protein being secreted. The conditioned medium was then collected, and secreted protein levels were compared to non-stimulated controls as well as to primary-only and secondary-only stimulation conditions.

Our results indicate that exposure to primary or secondary stimulation alone results in higher levels of osteocalcin protein compared to non-stimulated controls (Figure 2b; one-way ANOVA and post hoc Tukey test, *p* < 0.0001 for primary-alone vs. non-stimulated controls and *p* < 0.0001 for secondary-alone vs. non-stimulated controls). Exposure to both primary and secondary stimulation with a one-day rest in between resulted in significantly higher secreted osteocalcin levels compared to single regimen control conditions and to non-stimulated controls (one-way ANOVA and post hoc Tukey test, *p* < 0.0001 for primary and secondary stimulation conditions (C4–C7) compared to non-stimulated controls). Finally, exposure to the C7 stimulation condition, which included a primary and secondary stimulation with a secondary stimulation frequency of 20 Hz, resulted in the highest levels of secreted osteocalcin protein compared to all other groups (one-way ANOVA *p* = 0.0001, and post hoc Tukey test, *p* < 0.0001 for C7 vs. C1, C2, C3, and C4, *p* = 0.0003 for C7 vs. C5, and *p* = 0.0086 for C7 vs. C6). Interestingly, the C7 condition had the highest frequency of secondary stimulation compared to all other conditions, suggesting that frequency may be an important factor in the regulation of osteocalcin secretion.

In contrast, alkaline phosphatase levels were significantly reduced when constructs were exposed to the secondary stimulation condition alone (C2) and were further decreased when only primary stimulation (C3) was applied compared to non-stimulated controls (C1) (Figure 2c, one-way ANOVA and post hoc Tukey test, *p* = 0.0151 for secondary-alone vs. non-stimulated controls, *p* = 0.0036 for secondary-alone vs. primary-alone conditions, and *p* < 0.0001 for primary-alone vs. non-stimulated controls). When both primary and secondary stimuli were applied to constructs, alkaline phosphatase levels were significantly decreased compared to non-stimulated controls, primary-only stimulation, and secondary-only stimulation in all conditions except the C6 stimulation condition, compared to the primary-alone (C3) condition (Figure 2c, one-way ANOVA and post hoc Tukey test *p* = 0.2079 for C3 vs. C6). The C6 secondary stimulation condition had a shorter rest time (4 s) compared to all other conditions. Shorter rest times in between electrical stimulation pulses had no effect on osteocalcin protein levels but did affect alkaline phosphatase levels. The C6 condition where the 10 Hz secondary stimulation rest time was only 4 s between pulses exhibited significantly higher levels of alkaline phosphatase in the medium compared to the 9 s rest time used in the C4 and C7 conditions, suggesting longer rest times (9 s) lower alkaline phosphatase protein secretion. However, the C5 condition had a longer total duration of stimulation (x_1_) at 2 s compared to all other groups total stimulation duration of 1 s and a long (8 s) rest time. As a result, alkaline phosphatase secretion in the C5 group was not significantly different from the C6 or C7 conditions, suggesting that increasing the duration of stimulation in combination with a longer rest time could lower alkaline phosphatase secretion levels. The groups with the lowest levels of secreted alkaline phosphatase were C4 (primary and secondary stimulation with a secondary stimulation frequency of 10 Hz with a 9 s rest time, one-way ANOVA, and post hoc Tukey test, *p* < 0.0001 for C4 vs. C1, C2, C3, and C6, *p* = 0.0002 for C4 vs. C5, and *p* = 0.1267 for C4 vs. C7) and C7 (primary and secondary stimulation with a secondary frequency of 20 Hz with a 9 s rest time, one-way ANOVA and post hoc Tukey test, *p* < 0.0001 for C7 vs. C1, C2, and C3, *p* = 0.1267 for C7 vs. C4, *p* = 0.0895 for C7 vs. C5, and *p* = 0.0018 for C7 vs. C6) (Figure 2c). Interestingly, both C4 and C7 had the same total duration of stimulation (x_1_) and total rest time (x_2_) but differed in their pulse width (x_3_) and calculated frequency, suggesting that rest time rather than frequency may be the most important parameter for lowering alkaline phosphatase.

### 3.2. Electrical Stimulation of 3D Osteoblast Cell Culture Alters RNA Expression

**Figure 3:**
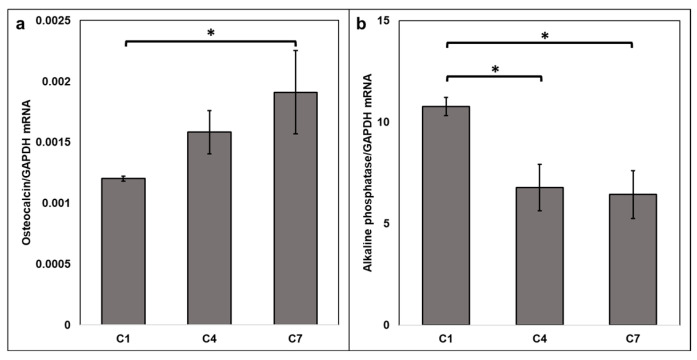
Electrical stimulation affects osteocalcin and alkaline phosphatase gene expression. (**a**) Significantly higher osteocalcin mRNA levels in constructs from stimulation condition C7 as compared to non-stimulated controls. (Kruskal–Wallis * *p* = 0.036, post hoc Dunn test between control C1 vs. C7 groups * *p* = 0.045). Samples were measured in triplicate. (**b**) Alkaline phosphatase mRNA levels were significantly lower in bone constructs from stimulation conditions C7 and C4 compared to non-stimulated controls (C1). One-way ANOVA ** *p* = 0.009; post hoc Dunn test C1 vs. C4 * *p* = 0.015 and C1 vs. C7 * *p* = 0.012. Osteocalcin and alkaline phosphatase mRNA levels were normalized to GAPDH, which did not vary between conditions. Error bars represent SEM. This experiment was repeated 4 times for a total n of 12–15/group.

To determine how changes in electrical stimulation parameters such as duration, rest time, and frequency impact alkaline phosphatase and osteocalcin gene expression, mRNA levels were quantified. Stimulation conditions C4 and C7 (with a secondary stimulation frequency of 10 Hz and 20 Hz, respectively) were selected for gene expression experiments because they showed the greatest differences in protein expression compared to non-stimulated controls (C1) for both targets. Osteocalcin mRNA levels were significantly higher in the C7 group where the secondary stimulation frequency was 20 Hz compared to non-stimulated controls (Kruskal–Wallis test, *p* = 0.036; post hoc Dunn test between control C1 and C7 groups, *p* = 0.045, and between C4 and C7, *p* = 1) (Figure 3a). Additionally, alkaline phosphatase mRNA levels were significantly lower under stimulation conditions C4 (10 Hz) and C7 (20 Hz) compared to non-stimulated controls, which is consistent with what was observed at the protein level (one-way ANOVA, *p* = 0.009) (Figure 3b). A post hoc Dunn test revealed that C4 and C7 conditions were not significantly different from each other.

### 3.3. ECM Composition Regulates Osteocalcin Secretion

**Figure 4:**
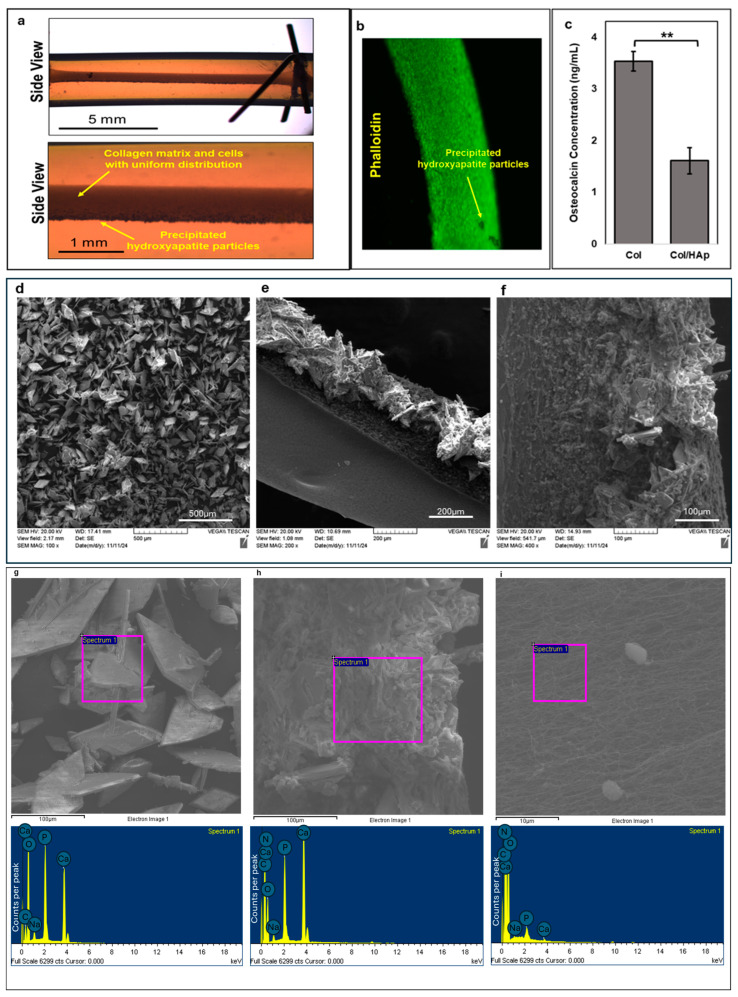
Hydroxyapatite added to collagen ECM creates an in vivo like microenvironment. (**a**) Non-uniform distribution of hydroxyapatite particles in the constructs (low- and high-magnification construct images using the Infinity Optical System microscope from top and bottom panels in a side view), (**b**) uniform distribution of cells despite non-uniformity of hydroxyapatite (HA) particles shown by staining cells with phalloidin for actin, (**c**) collagen-only constructs resulted in significantly higher levels of osteocalcin protein secreted into the medium compared to collagen and HA combined constructs. Student’s *t*-test ** *p* = 0.005. *n* = 3, Col: collagen-only construct, Col/HAp: construct with both collagen and HA as their ECM. Error bars represent standard error of the mean. (**d**) Scanning electron microscopy (SEM) image of HA alone (100×). (**e**) SEM image of the precipitated HA particles in the 3D construct (200×). (**f**) SEM image of HA particles precipitated to one side of the construct with bone cells and ECM on and around them (400×). (**g**) % Weight of elements using energy-dispersive spectroscopy (EDS). HA powder was used as a positive control to confirm composition. The main components in HA are phosphorous and calcium. Samples containing HA showed significantly elevated levels of these two elements compared to samples without HA. (**g**) HA powder with % weight (below) showing high levels of calcium and phosphorous. (**h**) Sample containing HA shows high levels of calcium and phosphorous. (**i**) Sample with no HA shows little to no calcium and phosphorous.

Collagen was used in the current method as the base ECM, but to mimic the calcium phosphate component of bone tissue, hydroxyapatite particles were included in the construct. Since these particles had higher density than the original collagenous bioink, they agglomerated and precipitated before the collagen gelled, resulting in their non-uniform distribution in the constructs (Figure 4a). Despite this non-uniform distribution, cells were still able to form constructs resembling those without hydroxyapatite in terms of general shape and size (Figure 4b). Constructs were maintained in culture similar to dynamic condition C4 based on protein secretion results, and their osteocalcin levels were compared to constructs grown in the same condition but without hydroxyapatite. Osteocalcin levels were significantly lower (Student’s *t*-test, *p* = 0.005) in the conditioned medium when hydroxyapatite was used than when it was absent (Figure 4c), likely due to osteocalcin binding to the hydroxyapatite particles. Using scanning electron microcopy and energy-dispersive spectroscopy (EDS), we confirmed that the precipitate in the hydroxyapatite containing constructs was, in fact, hydroxyapatite and not another ECM component or collagen. Hydroxyapatite has a unique shape (Figure 4d–f) with a sharp crystalline structure compared to collagen. Using EDS, we found that samples containing hydroxyapatite had a significantly higher percent weight for calcium and phosphorous, the two major components of hydroxyapatite, compared to samples that did not have hydroxyapatite (Figure 4g–i).

### 3.4. Electrical Stimulation of 3D Osteoblast Cell Cultures Alters Calcium Deposition

**Figure 5:**
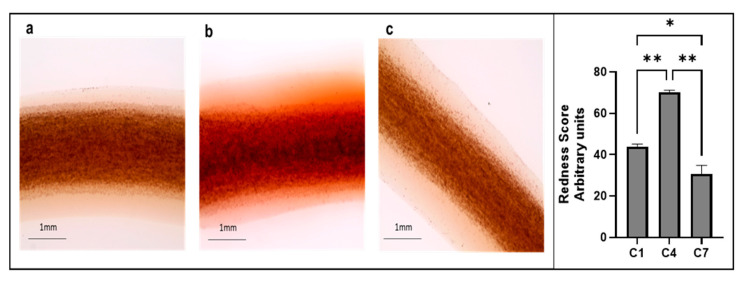
Alizarin Red staining of 3D constructs shows significant differences in calcium deposition. (**a**) Bone cell constructs from the C1 condition stained with Alizarin Red; (**b**) bone cell constructs from the C4 condition stained with Alizarin Red; (**c**) bone cell constructs from the C7 condition stained with Alizarin Red. One-way ANOVA, *p* = 0.0015 (Tukey multiple comparisons C1 vs. C4; ** *p* = 0.0046; C1 vs. C7, * *p* = 0.03; and C4 vs. C7, ** *p* = 0.0014). Images were taken using the EVOS Cell Imaging System. *n* = 2/group. Error bars represent the standard deviation.

Alizarin Red staining was used to measure calcium deposition in the constructs for conditions C1, C4, and C7 (Figure 5). Of all conditions analyzed, C4 had the highest deposition of calcium at the end of the 8 days of stimulation compared to no-stimulation (C1) controls and the C7 condition which had a higher frequency of stimulation (one-way ANOVA, *p* = 0.0015; Tukey multiple comparisons test C1 vs. C4, *p* = 0.005, and C7 vs. C4, *p* = 0.0014). Interestingly, C1 had the second highest level of calcium (C1 vs. C7, *p* = 0.03). These results suggest that higher frequencies may lower calcium deposition.

## 4. Discussion

Three-dimensional dynamic in vitro models are better representatives of in vivo conditions and more accurately resemble physiological activities compared to 2D static models [31,35]. Here, a previously developed biofabrication technique [30] was used to construct 3D models of human bone tissue. Electrical stimulation was applied to create a dynamic microenvironment for the SaOS-2 human osteosarcoma cells used in this study. We found that similar to exercise, which can elicit changes in osteocalcin and alkaline phosphatase levels, electrical stimulation of the 3D bone cell constructs can also alter osteocalcin and alkaline phosphatase levels.

In the current study, the electrical stimulation of 3D SaOS-2 bone constructs elicited changes to osteocalcin and alkaline phosphatase at both the mRNA and protein levels. Specifically, osteocalcin protein secretion was significantly increased when samples were electrically stimulated compared to non-stimulated controls. The condition that included both a primary and secondary stimulation at the highest frequency and the longest rest time (x_2_) exhibited the highest levels of secreted osteocalcin in the medium compared to all other conditions. Osteocalcin transcription was also significantly increased in the same high frequency/long rest time group compared to non-stimulated controls. Since stimulation with a frequency of 20 Hz increased osteocalcin compared to 10 Hz with the same rest time (9 s), this indicates that the frequency of the electrical stimulation may be the most important factor for increasing osteocalcin levels.

Conversely, alkaline phosphatase secreted protein and mRNA levels were significantly decreased with stimulation compared to non-stimulated controls, regardless of frequency. Alkaline phosphatase protein secretion was further decreased when constructs were stimulated with both primary and secondary regimens compared to primary or secondary stimulation alone, except in the case of the C6 condition which had a short rest time (x_2_ = 4 s). This result suggests that the rest time is important for alkaline phosphatase regulation and that shorter rest times in between stimulation cycles can result in more alkaline phosphatase secretion.

Alkaline phosphatase is an early marker of bone differentiation [23,36] and is highly expressed during the early stages of osteoblast cell development. Osteocalcin, on the other hand, is a late marker of bone differentiation [23,37] and is found at higher levels during later stages of osteoblast maturation, indicating a more adult-like cell phenotype. In this 3D model the effects of timing, rest time, intensity, and frequency of electrical stimulation resulted in significant increases in osteocalcin and decreases in alkaline phosphatase protein secretion and gene expression. These findings suggest that the cells represent a more mature cellular state compared to non-stimulated controls. Applying electrical stimulation that consists of both a primary and secondary stimulation pattern to 3D cultures of osteoblast-like cells may enable them to reach a more mature cell phenotype commonly found in adult bone cells. This can increase the physiological relevance of the model for studying adult bone tissue.

One possible mechanism explaining how electrical stimulation can increase osteocalcin mRNA expression and protein secretion is through an inverse piezoelectric effect [38]. Electrical stimulation has been shown to improve bone regeneration after damage. A previous study found that electrical stimulation caused a strain/stress effect on the bone through the inverse piezoelectric effect triggering bone cell stimulation and growth, which also increases osteocalcin secretion [33,39]. This process activates an internal electrical signal that increases bone remodeling and new bone formation. Another possible mechanism for the increase in secreted osteocalcin levels is that electrical stimulation can increase the release of proteins from bone which are known to increase osteocalcin levels [40]. For example, a study found that interleukin 6 (IL-6), a cytokine that is important for cell signaling and is produced in many tissues including bone and skeletal muscle, is released from tissues after electrical stimulation. Cytokines such as IL-6 are known to increase osteocalcin levels.

One possible reason for the decrease in alkaline phosphatase could be the release of different factors from bone under these specific stimulation conditions that act directly to inhibit alkaline phosphatase transcription. For example, bone secretes factors which are known to downregulate alkaline phosphatase expression through FGFR1/3-mediated activation of ERK1/2 [41]. It is possible that when stimulated, the mature osteoblast-like 3D bone cell constructs secrete alkaline phosphatase down-regulating factors, inhibiting alkaline phosphatase mRNA expression.

Collagen, the most abundant protein in bone tissue [40], was used in the current model as the base ECM. The inorganic phase of bone tissue, which is also an abundant component of bone, is mainly composed of calcium and phosphate ions. Together, these ions form hydroxyapatite crystals that are not only important for defining the mechanical resilience of bone, but they also play a major role in bone’s other functions as a source of sequestration and a reservoir for the secretion of factors involved in both autocrine and endocrine signaling [42,43]. To create a physiologically relevant model, hydroxyapatite particles were included in the construct to mimic the inorganic phase of the tissue. Osteocalcin has a high affinity for calcium ions in hydroxyapatite, where it binds to regulate bone mineralization [44]. Interestingly, we found that the secretion of osteocalcin from constructs was significantly reduced by the presence of hydroxyapatite in the tissue model. This highlights the importance of recreating features of in vivo systems that reflect physiologically relevant in vitro models. Additionally, constructs stained with Alizarin Red following electrical stimulation demonstrated that constructs subjected to primary and secondary stimulation at 10 Hz exhibited the highest levels of calcium deposition, constructs with no stimulation had the second highest calcium deposition, and constructs stimulated with secondary stimulation at 20 Hz showed the lowest calcium deposition. Many studies have found that electrical stimulation increases calcium deposition. One study showed an increase in intracellular calcium after electrical stimulation [45]. However, our study demonstrates that calcium deposition may be frequency dependent.

Our findings are consistent with cell culture and exercise studies in humans examining changes in osteocalcin. However, they are less consistent with studies examining alkaline phosphatase [46,47,48]. In an exercise study in humans, it was reported that High Intensity Interval Exercise (HIIE) increased osteocalcin serum levels in both men and women (ages ranged between 18 and 45 *y*/*o*) when tested immediately after exercise compared to 48 h after exercise [46]. Similarly, a study found that one session of HIIE was enough to increase serum uncarboxylated osteocalcin levels (a form of osteocalcin released from bone into the circulatory system) in healthy 20–26-year-old, sedentary males [49]. Another study found aerobic exercise significantly increased osteocalcin and alkaline phosphatase in inactive men after an 8-week workout period [48]. A study examining the effects of different types of electrical stimulation on SaOS-2 cells found that all forms of electrical stimulation increased both osteocalcin and alkaline phosphatase [50]. However, not all studies found that alkaline phosphatase levels increased significantly after exercise in humans. Alkaline phosphatase is a protein that is found in almost every tissue in the body and has many different isoforms. One study found that different isoforms of alkaline phosphatase responded differently to different types of exercise in women of different ages [51]. Specifically, levels of alkaline phosphatase L1, a liver isoform, decreased significantly in the serum of eight healthy postmenopausal women after ergometer cycling, while levels of bone-derived alkaline phosphatase isoforms increased. While one bone-derived alkaline phosphatase isoform was significantly increased in young healthy women after 30–40 min of jogging, all other isoforms and total alkaline phosphatase remained unchanged [51]. This example highlights that future work should examine changes in alkaline phosphatase at the isoform level and not just total alkaline phosphatase, as in the current study.

There are several possible reasons why we do not see the same reported changes in alkaline phosphatase as those found in some of the literature. The type and intensity of stimulation can differentially regulate gene expression. For example, osteocalcin and alkaline phosphatase levels increased with different modes of mechanical stimulation including compression, stretch, and shear stress, in both 2D and 3D cultures [52]. However, application of a pulsed electromagnetic field on osteoblasts during the mineralization stage decreased bone tissue-like formation (differentiation of stem cells to osteoblasts) and alkaline phosphatase activity [53]. Cells grown on conductive scaffolds and exposed to direct electrical stimulation showed an increase in both osteocalcin and alkaline phosphatase when electrical stimulation intensity was 200 mV/mm, whereas both of these markers decreased when the intensity was increased to 400 mV/mm [54]. The current study found that electrical stimulation increased osteocalcin and decreased alkaline phosphatase depending on the type of electrical stimulation and parameters applied. This may be due to differences in experimental design and types of electrical stimulation, and frequencies used compared to other studies. There are also significant differences in the length of stimulation exposure in each of these studies compared to our study. Cells grown on conductive scaffolds were exposed to electrical stimulation for a maximum of 8 h and then allowed to rest for 48 h before samples were harvested. Our treatment exposure was modeled to resemble regular activity experienced by bone over a prolonged period of 8 days. This prolonged exposure may induce changes in alkaline phosphatase regulation. Additionally, changes in transcription and protein levels can occur rapidly after stimulation, and therefore the decision to harvest our samples directly after electrical stimulation to provide an immediate assessment of its effects was preferred. This in vivo-like model can be used in future studies to accurately model multi-cell and tissue systems, for example, to study bone cell involvement in the secretion of hormonal and growth factors that are involved in physiological processes in other tissues, including adipogenesis, neuronal development, and muscle growth [24].

While elucidating the signal transduction pathways that are activated when bone cells are electrically stimulated was outside the scope of this study, previous studies have investigated this. One study in particular reported that electrical stimulation using capacitive and inductive coupling combined activated signal transduction through the intracellular release of Ca^2+^ which led to increases in cytosolic Ca^2+^ and increased cytoskeletal calmodulin activation [45]. This type of combined capacitive and inductive electrical stimulation is similar to the electrical stimulation used in the current study, and this may explain why increases in intracellular Ca^2+^ occur here. Future mechanistic studies of this specific model are needed to provide more insight into the downstream pathways that are activated and how they affect osteocalcin and alkaline phosphatase expression and secretion in bone. Future studies should also investigate other targets such as bone morphogenetic protein, bone sialoprotein, osteopontin, and collagen I to obtain a more detailed picture of the response of 3D bone constructs to electrical stimulation.

Electrical stimulation can potentially be used for recreating the effect of exercise on bone cells in vitro by affecting expression and secretion levels of osteocalcin and alkaline phosphatase. Electrical stimulation provides a tool that can stimulate expression of various key biomarkers that influence other organs in the body and mediate the influence of exercise on them. Future studies should also utilize this dynamic 3D bone cell model to study bone as an endocrine organ to determine how electrically stimulating bone cells and the resulting changes in osteocalcin and alkaline phosphatase levels can affect other molecules in other tissues in the body. For example, osteocalcin, when elevated in the periphery, increases levels of brain derived neurotrophic factor (BDNF), a molecule important for learning and memory, in the hippocampus of aged mice [47]. Many such studies suggest that bone and its derived hormones and molecules can affect other organs and tissues in the body, and the ability to regulate their expression levels using a biophysical stimulus can provide a potential unique therapeutic tool.

## 5. Conclusions

We developed a 3D model of bone tissue with a physiologically relevant ECM and dynamic microenvironment that uses electrical stimulation as a means to control the osteoblast cell function and phenotype. Alkaline phosphatase and osteocalcin were used to describe the osteoblast response to different electrical stimulation and culture conditions. Although electrical stimulation in in vitro and in vivo models is known to induce differentiation to the osteoblastic phenotype, its effect on already differentiated osteoblasts in 3D culture has been studied less. Here, it was shown that electrical stimulation of 3D bone constructs using the osteoblast SaOS-2 cell line directly affects osteoblast mRNA and protein expression. Specific electrical stimulation, frequency, duration, and rest time parameters in our study increased osteocalcin protein secretion and decreased alkaline phosphatase protein secretion. We showed that the amount of increase or decrease is frequency-, duration- and intensity-dependent. Our model shows that optimal conditions for increasing osteocalcin protein secretion and transcriptional upregulation require both a primary and secondary stimulation regimen with a secondary stimulation at a higher frequency. Alkaline phosphatase protein secretion and mRNA expression were reduced using a stimulation that included both a primary and secondary stimulation with a secondary stimulation total rest time being most important. These results indicate that electrical stimulation patterns and frequencies can be developed to optimize the production and release of different hormones and proteins from bone. It has been shown previously that the simultaneous application of electrical and mechanical stimuli can enhance the proliferation and differentiation of pre-osteoblasts as compared to only one stimulation mode [55]. The model developed here can be further improved by the simultaneous application of electrical and mechanical stimuli, which has been shown to be feasible using this platform [30]. The findings from this study may be used to gain further insight into how electrical stimulation frequencies can modify the levels of hormones and proteins secreted from bone to better understand bone as an endocrine organ and to improve overall health.

## Figures and Tables

**Table 1:** Sequences of qPCR primers.

Target and Accession Number	Forward Primer	Reverse Primer	PCR Product Size (bp)
*Human Alkaline Phosphatase* AH005272.2	AGGACGCTGGGAAATCTGTG	CATGAGCTGGTAGGCGATGT	164
*Human Osteocalcin* NM_199173	CTCACACTCCTCGCCCTATT	CGCCTGGGTCTCTTCACTAC	143
*Human GAPDH* NM_002046	TCTCCTCTGACTTCAACAGCGAC	CCCTGTTGCTGTAGCCAAATTC	126

## Data Availability

Data are available in McMaster Dataverse, a collection within Borealis as a publicly archived dataset (https://doi.org/10.5683/SP3/IJGYHA).
